# Sperm morphology indices as exploratory screening tools for elevated DNA fragmentation: a single-centre correlation and threshold derivation study

**DOI:** 10.1186/s12894-026-02261-8

**Published:** 2026-07-18

**Authors:** Abhishek Ramakrishnan, Peter Osvath, Rudolf Gesztelyi, Csaba Csikos, Bela Juhasz, Aniko Pósa, Miklos Szücs

**Affiliations:** 1 University of Debrecen, Egyetem tér 1, Debrecen, 4032 Hungary; 2 University of Szeged, Dugonics tér 13, Szeged, 6720 Hungary

**Keywords:** Sperm morphology, Teratozoospermia Index, Multiple Anomalies Index, Sperm Deformity Index, DNA fragmentation index, ROC analysis, Sperm chromatin dispersion

## Abstract

**Background:**

Sperm morphology assessment remains a routine component of semen analysis, yet its relationship with sperm DNA fragmentation at the level of individual morphological indices is incompletely characterised. This study evaluated correlations between the Teratozoospermia Index (TZI), Sperm Deformity Index (SDI), and Multiple Anomalies Index (MAI) with the DNA Fragmentation Index (DFI), deriving exploratory thresholds to identify men at risk of elevated DNA fragmentation (DFI > 20%).

**Methods:**

A retrospective analysis was conducted on 135 semen samples from men aged 20–55 years attending a university andrology centre. Morphology was assessed on Papanicolaou-stained smears; TZI, SDI, and MAI were calculated manually. DNA fragmentation was measured using the GC-SCD-20 kit. Pearson correlation and receiver operating characteristic (ROC) analysis with Youden-optimised thresholds were performed.

**Results:**

All three indices showed statistically significant, weak positive correlations with DFI: TZI (*r* = 0.263, *p* = 0.001), SDI (*r* = 0.290, *p* = 0.004), and MAI (*r* = 0.371, *p* < 0.001). ROC analysis demonstrated modest discriminatory performance: AUC values of 0.645, 0.620, and 0.680 respectively. At thresholds of TZI > 1.85, SDI > 2.42, and MAI > 2.53, sensitivity ranged from 82% to 90% with specificity of 44–53%.

**Conclusion:**

TZI, SDI, and MAI correlate weakly but significantly with DFI and demonstrate a high-sensitivity, low-specificity screening profile. These thresholds are exploratory, derived from a single cohort, and require prospective validation in larger independent studies before clinical application. They should be regarded as adjunctive tools within routine semen analysis and cannot substitute for direct DNA fragmentation testing.

## Introduction

The sixth edition of the WHO Laboratory Manual for the Examination and Processing of Human Semen (2021) identifies sperm concentration, motility, vitality, and morphology as the core parameters of routine semen analysis [1]. Among these, morphology has historically been regarded as a predictor of fertilising potential, serving as an indirect indicator of spermatogenic efficiency [2]. Structural defects affecting the head, mid-piece, or tail have been linked mechanistically to impaired chromatin packaging, defective nuclear condensation, and increased susceptibility to DNA strand breakage [3, 4]. Since sperm DNA integrity is now recognised as an independent determinant of embryo development and assisted reproductive technology (ART) outcomes, understanding the quantitative relationship between morphological abnormalities and the DNA Fragmentation Index (DFI) carries direct clinical relevance [4, 5].

The DFI reflects the proportion of spermatozoa carrying nuclear DNA strand breaks. Elevated values arise from upstream insults including oxidative stress, defective apoptosis, aberrant protamination, and environmental exposures — many of which also disrupt normal sperm morphogenesis [5]. Despite these shared pathophysiological origins, the specific relationship between composite morphological indices and DFI has not been consistently established, in part because most prior work relies on the simple percentage of morphologically normal forms rather than on indices that capture the distribution and multiplicity of structural defects across the sperm population.

Several studies have demonstrated associations between elevated DFI and impaired semen parameters including reduced motility and abnormal morphology, with DFI now recognised as a clinically useful adjunct to conventional semen analysis in predicting ART outcomes [6–8]. Multiple assay platforms have been developed to quantify sperm DNA damage, each capturing distinct dimensions of chromatin integrity [9].

Three such indices have been developed for this purpose. The Sperm Deformity Index (SDI) is calculated as the total number of defects across all evaluated spermatozoa divided by the total number of cells, expressing the mean defect burden per spermatozoon in the full sample [10]. The Teratozoospermia Index (TZI) is restricted to morphologically abnormal cells and calculates the mean number of defect categories — head, mid-piece, tail, and cytoplasmic residue — per abnormal spermatozoon, capturing the structural complexity of defects among the abnormal fraction [10–11]. The Multiple Anomalies Index (MAI) also focuses on abnormal spermatozoa but counts all individual discrete defects rather than categories, offering a finer-grained quantification of per-cell morphological burden [11–12]. Each index captures a distinct dimension of sperm morphological pathology, and their relative informativeness with respect to DNA fragmentation has not been directly compared within a single cohort.

The clinical relevance of morphology-based assessment has evolved alongside the development of functional assays. The hyaluronan binding assay (HBA), which exploits sperm surface maturity characteristics as an indirect index of chromatin integrity [13], and composite indices such as the HBMaSC described by Szücs et al. [14] offer more mechanistically direct information than morphological scoring alone. However, these tests are not uniformly available across clinical settings, and standard semen morphology analysis continues to be performed routinely worldwide. Whether morphological indices can provide preliminary actionable information regarding DNA fragmentation risk — particularly where direct functional testing is limited — remains a clinically pertinent question.

This study had two objectives: first, to quantify correlations between TZI, SDI, and MAI and DFI in a cohort of men attending a single andrology centre for fertility evaluation; and second, to derive exploratory threshold values for each index that may identify men at elevated risk of clinically significant DNA fragmentation (DFI > 20%). All derived thresholds are intended as hypothesis-generating rather than prescriptive, and the study does not claim to establish clinical decision limits. These findings are presented as a quantitative basis for future prospective validation in larger, multi-centre cohorts.

## Materials and methods

### Study population

This was a single-centre retrospective analysis of semen samples collected at the University of Debrecen Andrology Centre between January 2024 and February 2025. Semen analysis, including assessment of sperm morphology and calculation of TZI, SDI, and MAI, together with sperm DNA fragmentation testing using the GC-SCD-20 sperm chromatin dispersion kit, are performed as part of routine clinical practice for all men attending the centre for fertility assessment, irrespective of baseline semen parameters. No samples were tested specifically for research purposes; all data were collected retrospectively from existing clinical records. All participants were men attending the centre for fertility assessment; none carried a confirmed diagnosis of male infertility at the time of sample collection. Men aged 20–55 years were eligible for inclusion. Exclusion criteria were: azoospermia; severe oligozoospermia (sperm concentration < 1 × 10⁶/mL); a documented history of chemotherapy or gonadotoxic radiotherapy; and known genetic disorders directly affecting spermatogenesis. Of approximately 200 men assessed at the centre during the study period, all of whom underwent the same standardised clinical protocol including DFI testing, 135 met the study eligibility criteria above and were included in the final analysis. As DFI testing was performed uniformly across all patients attending the centre rather than selectively ordered, the risk of selection bias related to DFI testing indication is considered minimal; the exclusions applied related solely to the predefined eligibility criteria listed above.

The study was conducted in accordance with the Declaration of Helsinki. All procedures complied with applicable institutional guidelines and were approved by the Institutional Review Board of the University of Debrecen (Approval No. DE RKEB.IKEB No. 7057 − 2024, 15 January 2024). Written informed consent was obtained from each participant prior to sample collection.

#### Population characteristics

Available demographic and clinical data for the study cohort are reported here. Mean sexual abstinence period prior to sample collection was 4.53 days, within the recommended range of 2–7 days specified by laboratory protocol. Mean body mass index (BMI) was 28.52 kg/m². All participants were non-smokers and reported no regular alcohol consumption. No participant had clinical or ultrasonographic evidence of varicocele, assessed by physical examination and scrotal ultrasound at the time of initial evaluation. Occupational exposure to gonadotoxic agents, environmental toxicants, or heat exposure was not formally assessed or recorded for this cohort, and is acknowledged as a limitation in the Discussion. Given that all participants were non-smokers, non-drinkers, and free of varicocele, these factors did not vary within the cohort and could not be evaluated as potential confounders in the statistical analysis, though this homogeneity also reduces the likelihood that these specific factors materially influenced the observed associations.

No formal sample size calculation was performed prior to data collection, as the study utilised all eligible samples available within the defined study period. The final cohort of 135 men is acknowledged as a limitation, and all findings should be interpreted as exploratory in nature.

### Sample collection and sample preparation

Semen samples were obtained by masturbation into sterile, wide-mouthed containers following 2–7 days of sexual abstinence. Immediately after collection, the samples were maintained at 37 °C and allowed to liquefy for 30–60 min before analysis.

Each specimen was gently mixed to ensure homogeneity, avoiding the introduction of air bubbles. During CASA analysis, an aliquot of liquefied semen was loaded into a pre-warmed disposable counting chamber maintained at 37 °C on a temperature-controlled microscope stage. During DNA fragmentation testing, a separate aliquot was processed using the GC-SCD-20 kit performed in accordance with the 6th edition of the WHO Laboratory Manual for the Examination and Processing of Human Semen [1].

### CASA system and semen parameters

All semen parameters were measured in accordance with WHO 6th edition (2021) criteria [1]. Analysis was performed using the Sperm Class Analyzer (SCA, Microptic S.L., Barcelona, Spain) [15], equipped with a phase-contrast microscope and high-speed digital camera, calibrated against manufacturer-supplied reference standards at the start of each analytical session. Sperm concentration (×10⁶/mL) and total sperm count per ejaculate were recorded automatically. Motility was classified as progressive (PR), non-progressive (NP), or immotile (IM), with associated kinematic parameters documented. Morphometric parameters generated by the SCA system were recorded but were not incorporated into the correlation analyses reported here.

### Morphological Indices (TZI, MAI, SDI)

Morphological evaluation was performed on Papanicolaou-stained smears examined under oil immersion at ×1000 magnification, with defect classification according to WHO (2021) criteria [1]. A minimum of 200 spermatozoa were assessed per sample, with defects affecting the head, mid-piece, tail, and cytoplasmic residue recorded individually for each cell. The three morphological indices were calculated as follows:

SDI (Sperm Deformity Index) = Total defects recorded across all evaluated spermatozoa divided by the total number of spermatozoa evaluated. This expresses the mean defect burden per cell across the entire sample population [10].

TZI (Teratozoospermia Index) = Total defect categories recorded among morphologically abnormal spermatozoa divided by the total number of morphologically abnormal spermatozoa. Each abnormal cell was scored across up to four defect categories (head, mid-piece, tail, cytoplasmic residue), reflecting the structural complexity of defects among the abnormal fraction [10–11].

MAI (Multiple Anomalies Index) = Total individual defects recorded among morphologically abnormal spermatozoa divided by the total number of morphologically abnormal spermatozoa. Unlike TZI, MAI counts all discrete defect instances rather than defect categories, yielding a finer quantification of morphological burden per abnormal spermatozoon [11–12].

All assessments and calculations were performed manually by a trained laboratory technician. To evaluate inter-observer reproducibility, 20% of slides were independently re-evaluated by a second trained observer blinded to the initial assessment. Concordance was expressed as percentage agreement in defect classification; only samples achieving ≥ 90% concordance were included in the final analysis, a threshold met across all included samples.

### Sperm DNA fragmentation

Sperm DNA fragmentation was assessed using the GC-SCD-20 sperm chromatin dispersion kit in accordance with the manufacturer’s instructions and consistent with WHO (2021) guidance [1]. Samples were washed with phosphate-buffered saline, resuspended in low-melting-point agarose at 37 °C, applied to pre-coated slides, and solidified at 4 °C. Slides were processed through sequential acid denaturation and nuclear lysis solutions to remove proteins and permit DNA loop relaxation, followed by graded ethanol dehydration and Diff-Quik staining. Prepared slides were analysed using the CASA system with integrated image analysis software. Spermatozoa were classified automatically by halo morphology: large or medium dispersion halos were scored as non-fragmented; small or absent halos as fragmented. A minimum of 500 spermatozoa were scored per sample. The DFI was expressed as the percentage of spermatozoa with fragmented DNA relative to the total counted. A clinical threshold of DFI > 20% was applied, consistent with published reference limits for sperm chromatin dispersion assays at which impaired reproductive outcomes have been reported [4]. 

### Quality control and data analysis

A minimum of 200 spermatozoa were analysed for morphology and at least 500 spermatozoa for DNA fragmentation per sample. All results were interpreted in accordance with WHO (2021) reference criteria. Quality control was maintained through routine CASA calibration, use of motility and morphometry standards, and periodic cross-validation of CASA-generated results and DFI scoring with manual evaluation by a trained laboratory technician.

### Statistical analysis

Data were analysed using IBM SPSS Statistics version 27.0 (IBM Corp., Armonk, NY, USA). Continuous variables are presented as mean ± standard deviation. Normality of TZI, SDI, MAI, and DFI distributions was confirmed using the Shapiro–Wilk test (*p* > 0.05 for all variables). Bivariate associations between each morphological index and DFI were assessed using Pearson’s correlation coefficient (r); a two-sided p-value of < 0.05 was considered statistically significant.

Receiver operating characteristic (ROC) curve analysis evaluated the ability of each index to identify men with DFI > 20%. Area under the curve (AUC) values with 95% confidence intervals were calculated. Optimal cut-off values were identified using Youden’s index (J = sensitivity + specificity − 1), which selects the threshold simultaneously maximising both sensitivity and specificity. Sensitivity and specificity at each optimal threshold were computed with 95% confidence intervals.

A multivariable logistic regression model incorporating TZI, SDI, or MAI alongside potential confounders (age, BMI, sperm concentration, total motility, and abstinence duration) was considered to assess whether each morphological index remains associated with DFI > 20% independently of these factors. However, this analysis was not performed in the present study. Reliable multivariable models generally require a sufficient number of patients with the outcome of interest (in this case, DFI > 20%) relative to the number of variables included, in order to avoid unstable or misleading results. Given the number of men with elevated DFI in this cohort relative to the number of clinically relevant variables that would need to be included, we judged that a multivariable model could not be estimated reliably at this stage. We therefore report bivariate correlations and ROC-derived thresholds as the primary analysis, consistent with the exploratory aim of this study. We agree that multivariable modelling is an important next step and recommend that future, larger studies prioritise this analysis to clarify the independent contribution of each index.

## Results

One hundred thirty five men with a mean age of 39.7 ± 7.0 years were included. Baseline semen parameters are summarised in Table 1. Mean sperm concentration was 37.43 ± 8.08 million/mL and total sperm count was 113.14 ± 44.55 million/ejaculate, both within the WHO reference range. Ejaculate volume averaged 3.47 ± 1.62 mL. Progressive motility was 38.62 ± 20.98%, non-progressive motility 11.28 ± 4.98%, and total motility 49.72 ± 21.27%, 50.10 ± 4.38% of spermatozoa classified as immotile. Normal morphology averaged 3.68 ± 1.57%, with abnormal morphology at 96.03 ± 3.11%. The mean DNA Fragmentation Index (DFI) was 22.62 ± 7.21%, exceeding the clinical threshold of > 20%, indicating that the cohort as a whole carried a meaningful burden of sperm DNA damage. Among the morphological indices, mean Teratozoospermia Index (TZI) was 1.92 ± 0.17, Sperm Deformity Index (SDI) was 2.64 ± 0.38, and Multiple Anomalies Index (MAI) was 2.87 ± 0.40, all exceeding their respective reference thresholds.

**Table 1:** Baseline semen parameters and DNA fragmentation (*N* = 135)

Sperm Parameters	Average ± SD	Reference range
Sperm concentration (million/ml)	37.43 ± 8.08	> 16 million/mL
Total sperm count (million/ ejaculate)	113.14 ± 44.55	> 39 million/ejaculate
Ejaculate Volume (ml)	3.47 ± 1.62	> 1.4 mL
Abstinence period (days)	4.50 ± 1.0	2–7 days
DNA Fragmentation Index (%)	22.62 ± 07.21	< 20%
Normal Morphology	3.68 ± 01.57	> 4%
Abnormal Morphology (%)	96.03 ± 3.11	-
Teratozoospermia Index	1.92 ± 0.17	< 4.0
Sperm Deformity Index	2.64 ± 0.38	< 3.0
Multiple Anomalies Index	2.87 ± 0.40	-
Acrosome Average (%)	45.43 ± 8.98	-
Acrosome Deviation (%)	14.81 ± 3.10	-
Progressive motility (%)	38.62 ± 20.98	> 32%
Non progressive motility (%)	11.28 ± 4.98	> 1%
Immotile (%)	50.10 ± 4.38	> 20%
Total motility (%)	49.72 ± 21.27	> 40%

All three morphological indices showed statistically significant positive correlations with DFI, though the strength of each association was weak. MAI demonstrated the strongest correlation (*r* = 0.371, *p* < 0.001), followed by SDI (*r* = 0.290, *p* = 0.004) and TZI (*r* = 0.263, *p* = 0.001). These findings indicate that greater morphological burden is associated with a higher proportion of spermatozoa carrying DNA strand breaks, regardless of whether burden is expressed as mean per-cell individual defect count, mean defect category count among abnormal cells, or mean defect load across the full sperm population. These are correlational observations; no causal inference can be drawn from this study design.

**Fig. 1:**
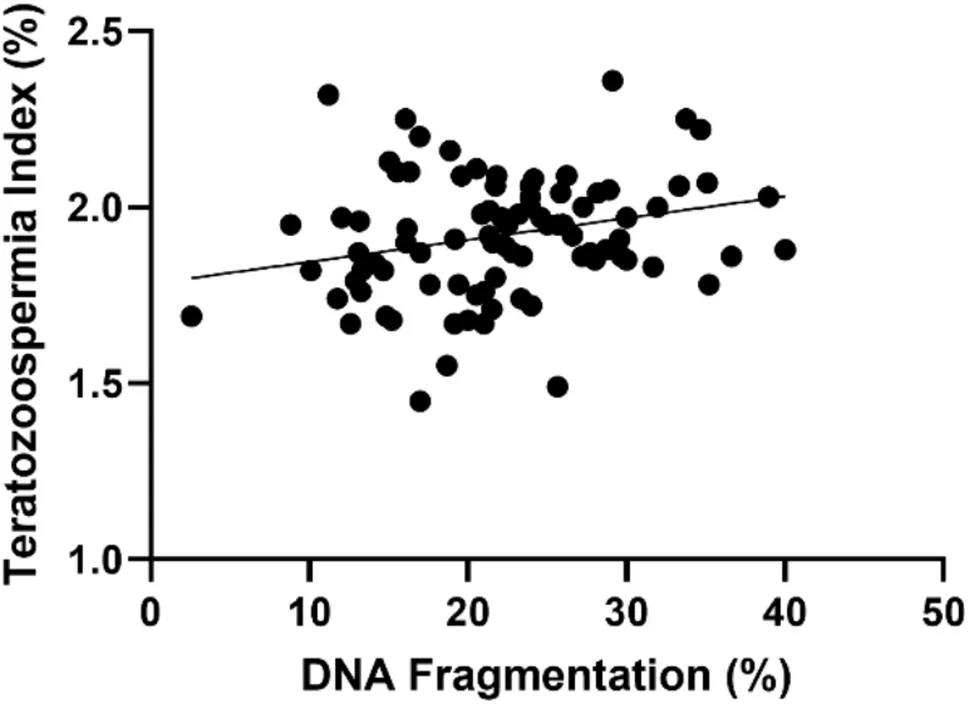
Scatter plot with linear regression line showing the correlation between Teratozoospermia Index (TZI) and DNA Fragmentation Index (DFI). The solid line represents the linear regression fit (*r* = 0.263, *p* = 0.001)

**Fig. 2:**
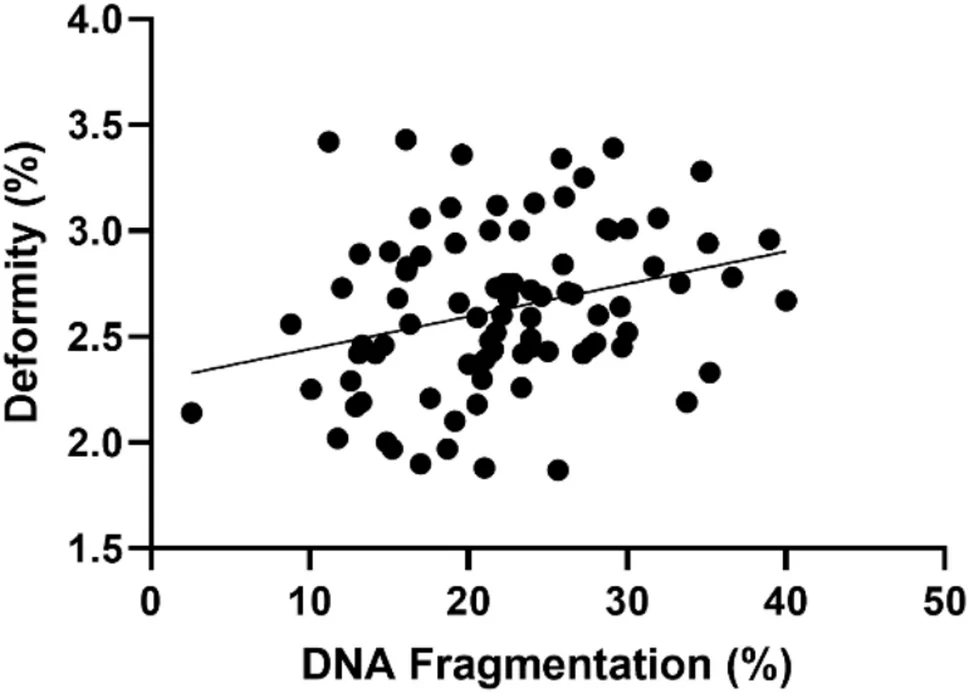
Scatter plot with linear regression line showing the correlation between Sperm Deformity Index (SDI) and DNA Fragmentation Index (DFI). The solid line represents the linear regression fit (*r* = 0.290, *p* = 0.004)

**Fig. 3:**
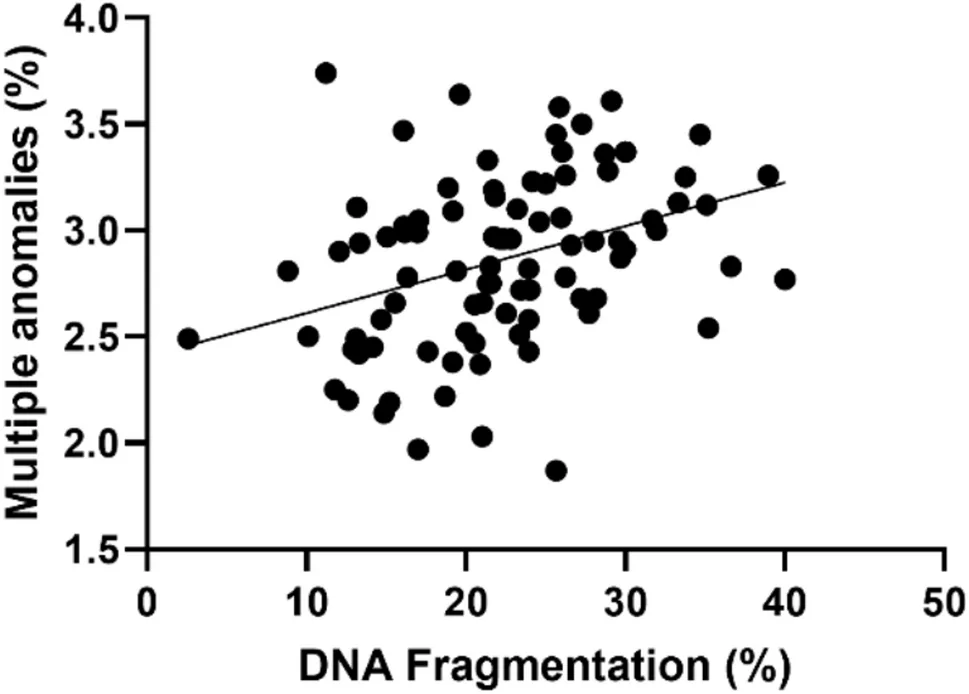
Scatter plot with linear regression line showing the correlation between Multiple Anomalies Index (MAI) and DNA Fragmentation Index (DFI). The solid line represents the linear regression fit (*r* = 0.371, *p* < 0.001)

Scatter plots with linear regression lines for TZI, SDI, and MAI against DFI are presented in Figs. 1 and 2, and 3 respectively. A small number of data points with high leverage were identified on visual inspection but were retained in all analyses, as their removal did not materially alter regression coefficients or Pearson r values.

**Fig. 4:**
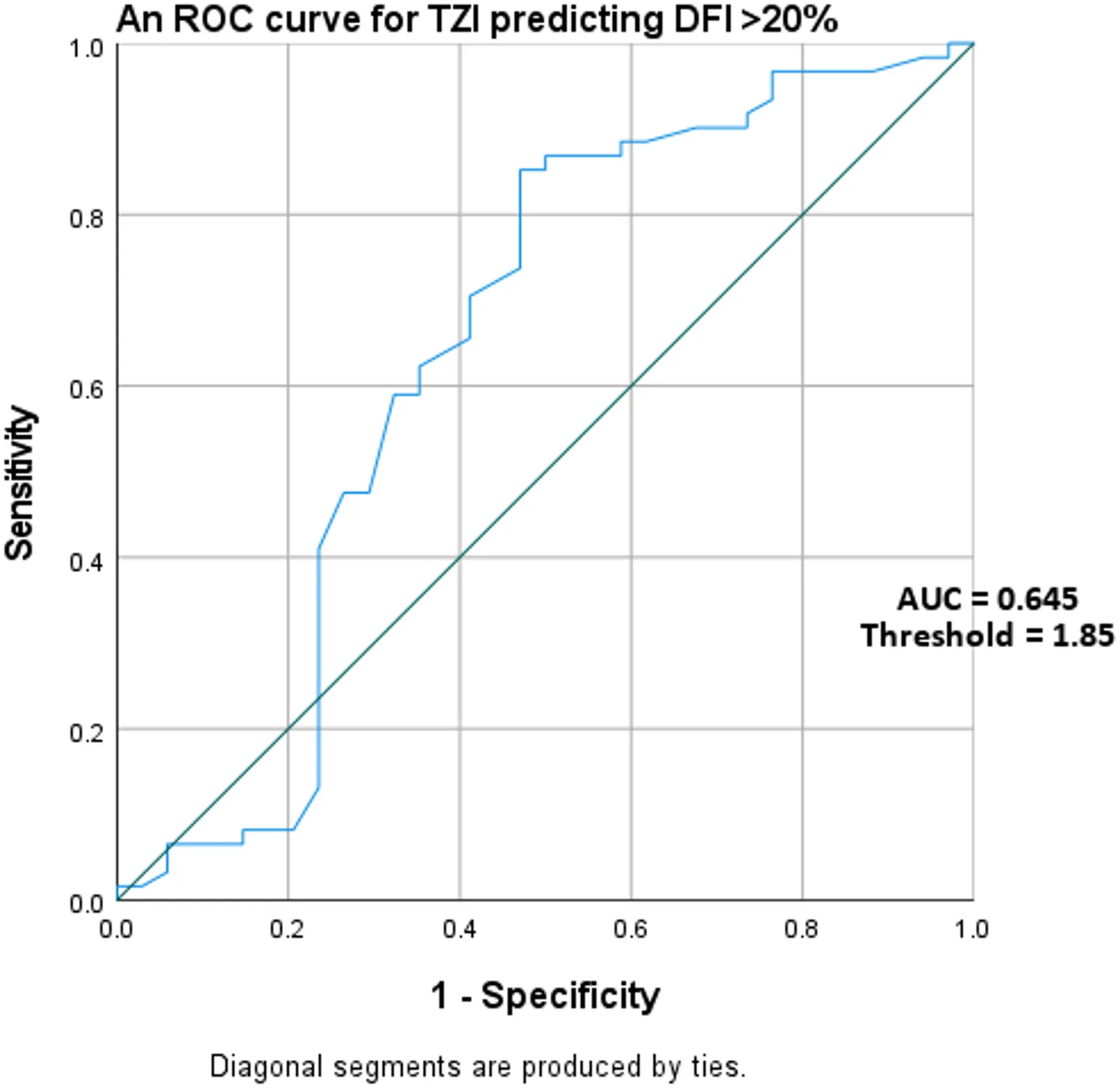
Receiver operating characteristic (ROC) curve for Teratozoospermia Index (TZI) in predicting DNA Fragmentation Index > 20%. AUC = 0.645 (95% CI: 0.54–0.75); optimal threshold TZI > 1.85

**Fig. 5:**
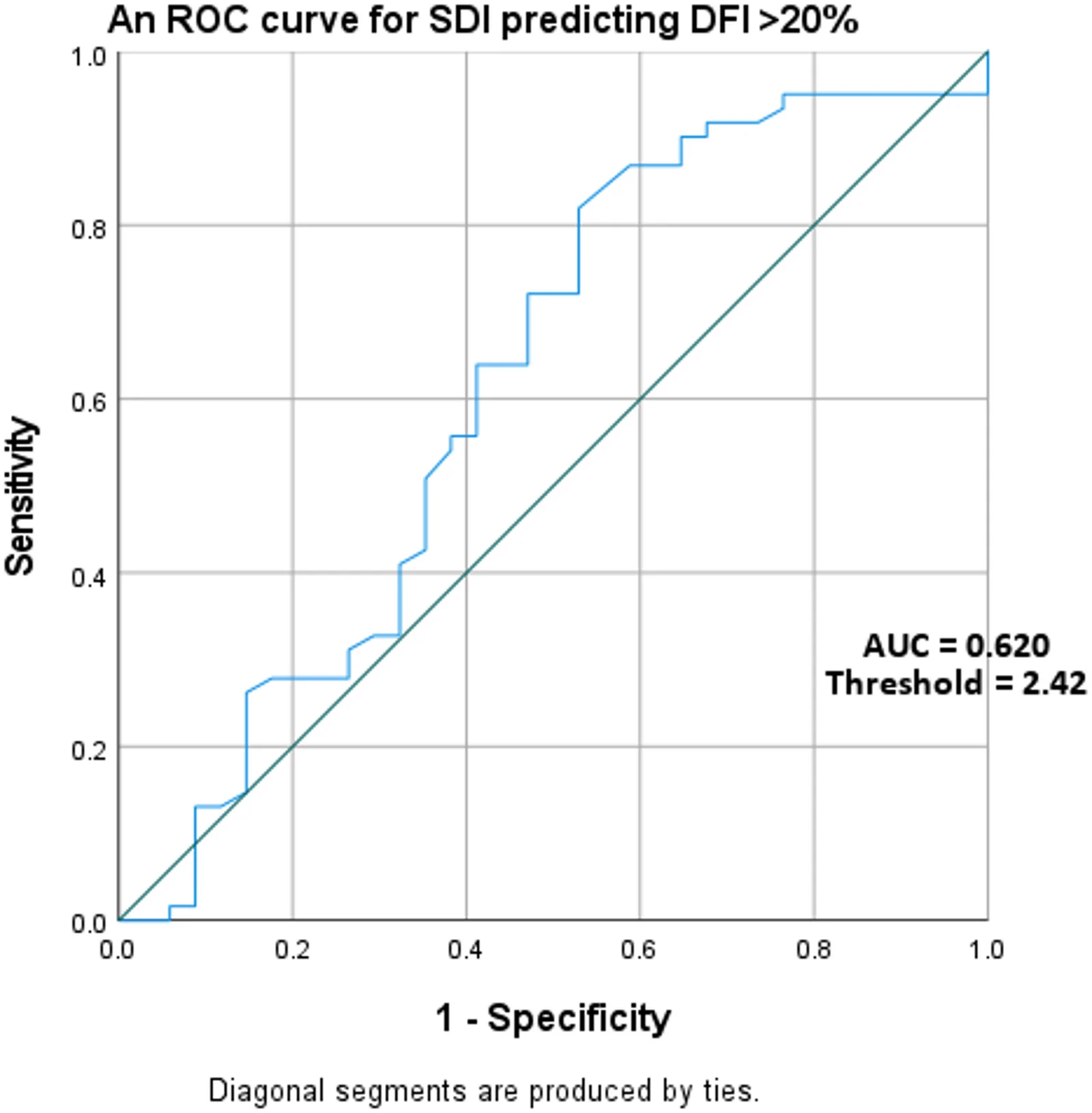
Receiver operating characteristic (ROC) curve for Sperm Deformity Index (SDI) in predicting DNA Fragmentation Index > 20%. AUC = 0.620 (95% CI: 0.50–0.74); optimal threshold SDI > 2.42

**Fig. 6:**
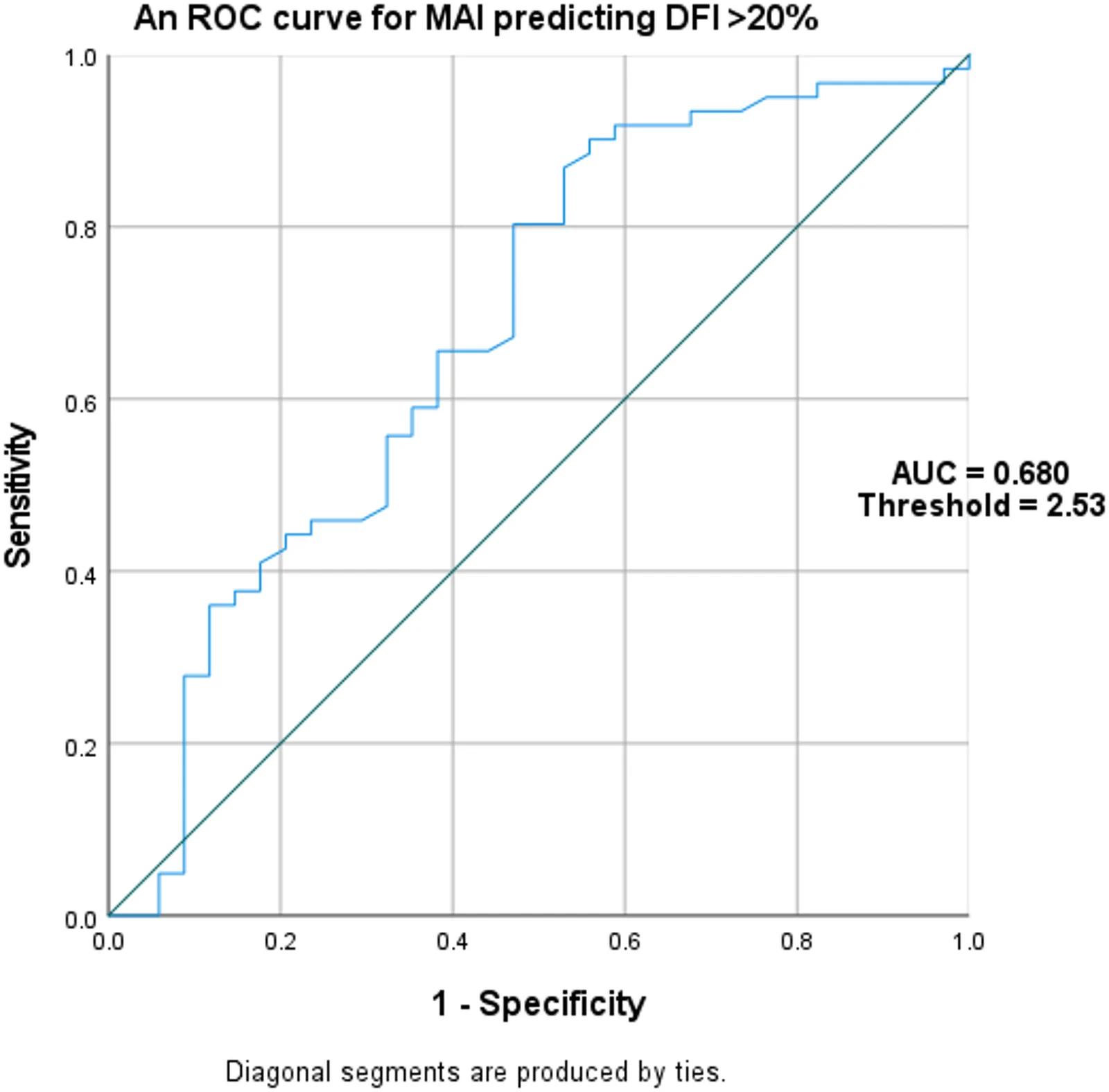
Receiver operating characteristic (ROC) curve for Multiple Anomalies Index (MAI) in predicting DNA Fragmentation Index > 20%. AUC = 0.680 (95% CI: 0.58–0.78); optimal threshold MAI > 2.53

ROC curve analysis assessed the discriminatory capacity of each index for identifying men with DFI > 20%. AUC values indicated modest performance throughout: MAI achieved the highest discriminatory ability (AUC = 0.680, 95% CI: 0.58–0.78), followed by TZI (AUC = 0.645, 95% CI: 0.54–0.75) and SDI (AUC = 0.620, 95% CI: 0.50–0.74). No index exceeded an AUC of 0.70, confirming limited capacity to reliably distinguish between elevated and non-elevated DFI status. ROC curves are displayed in Figs. 4 and 5, and 6.

Optimal thresholds identified by Youden’s index were: TZI > 1.85, SDI > 2.42, and MAI > 2.53. Sensitivity at these thresholds was consistently high — TZI: 82.1%, SDI: 82.0%, MAI: 90.2% — while specificity was uniformly low: TZI: 52.9%, SDI: 47.1%, MAI: 44.1%. This pattern reflects a screening-type performance profile: few men with elevated DFI are likely to be missed, but a considerable proportion of men without elevated DFI would be incorrectly identified as at risk. Full correlation and ROC data are presented in Table 2.

**Table 2:** Pearson Correlation and ROC analysis of sperm morphology indices with DFI > 20%

Index	*r* (*p* value )	AUC(95% CI)	Cut-off	Sensitivity (%)	Specificity (%)
TZI	0.263 (*p* = 0.001	0.645 (0.54–0.75)	1.85	82.1	52.9
SDI	0.290 (*p* = 0.004)	0.620 (0.50–0.74)	2.42	82.0	47.1
MAI	0.371 (*p* < 0.001)	0.680 (0.58–0.78)	2.53	90.2	44.1

These cut-off values were derived internally from the present cohort and represent exploratory thresholds. They have not been validated in an independent sample and should not be applied as clinical decision limits without prospective confirmation.

## Discussion

The principal finding of this study is that TZI, SDI, and MAI each correlate positively with DFI, though the associations are weak and the derived thresholds demonstrate only modest discriminatory performance. These results are consistent with the view that sperm morphology and DNA integrity share certain upstream determinants — principally defective spermatogenesis and oxidative stress — while remaining biologically distinct constructs that do not correspond reliably enough to permit one to serve as a clinical proxy for the other.

Among the three indices, MAI showed the strongest correlation with DFI (*r* = 0.371), followed by SDI (*r* = 0.290) and TZI (*r* = 0.263). The stronger correlation of MAI with DFI compared to TZI and SDI may reflect the fact that MAI captures a greater number of individual defect instances per abnormal spermatozoon, potentially providing a more granular representation of overall morphological deterioration. However, this does not establish a direct biological mechanism linking MAI specifically to DNA damage, and this interpretation should be regarded as hypothesis-generating rather than conclusive. Spermatozoa carrying multiple concurrent structural defects are thought to arise from more pronounced disruptions during spermatogenesis, which may simultaneously compromise chromatin compaction and protamination [16–18]. The absolute differences in correlation coefficients between the three indices are small, and designating MAI as a clinically superior parameter on the basis of this single exploratory dataset would be premature.

While the correlations observed are weak, they are consistent with shared upstream spermatogenic disruptions that simultaneously affect both morphological development and chromatin integrity. The biological pathways most plausibly underlying this association include the following. During spermiogenesis, nuclear DNA undergoes progressive condensation through sequential replacement of histones by transition proteins and subsequently by protamines [19]. Disruption of this process — from oxidative stress, defective apoptotic signalling, or intrinsic spermatogenic failure — can produce concurrent structural morphological defects and residual nuclear DNA strand breaks [20]. Reactive oxygen species generated at the mid-piece and mitochondrial level, particularly in spermatozoa with flagellar or cytoplasmic defects, may further amplify DNA damage through oxidative mechanisms [3, 21]. These overlapping aetiological pathways provide a mechanistic basis for the weak correlations observed while also explaining their lack of clinical precision: not all morphologically abnormal spermatozoa carry fragmented DNA, and morphologically normal cells may harbour DNA damage arising through pathways that produce no visible structural defect [19, 22].

The ROC analysis confirmed that none of the three indices is suitable as a primary diagnostic test for elevated DNA fragmentation. AUC values of 0.620 to 0.680 represent performance only marginally above chance, and specificity values of 44–53% at Youden-optimised thresholds indicate a high false-positive rate in practice. A substantial proportion of men without elevated DFI would be incorrectly identified as at risk — a burden that would drive unnecessary downstream investigation if these thresholds were applied without due consideration of their limitations. At the same time, sensitivity values of 82–90% suggest that few men with genuinely elevated DFI would be missed. This high-sensitivity, low-specificity profile is characteristic of a first-tier screening instrument rather than a confirmatory test, and it is in this more constrained role — as a preliminary triage tool in settings where direct DFI testing is limited — that morphological indices may retain some practical value.

The clinical scope of this application should be clearly bounded. Direct sperm DNA fragmentation testing by sperm chromatin dispersion, TUNEL, SCSA, or Comet assay remains the reference standard for chromatin integrity assessment [5]. The indices evaluated here cannot substitute for direct measurement, and the derived thresholds should not be used to defer or replace DFI testing when clinically indicated. Where direct testing is accessible, the incremental value of additionally computing TZI, SDI, or MAI for DFI risk stratification appears limited given the weak correlations and AUC values below 0.70. In resource-constrained environments, the high-sensitivity profiles observed may support their use as a preliminary flag for prioritised DFI evaluation, provided clinicians remain aware of the associated false-positive burden.

It is also worth noting that the mean DFI of the present cohort was 22.62 ± 7.21%, marginally exceeding the applied clinical threshold of > 20%. This suggests that the study population represented a group with borderline to mildly elevated DNA fragmentation rather than severe chromatin damage, which may have attenuated the correlations observed. Whether morphological indices perform differently in cohorts with more pronounced DFI elevation warrants investigation in future studies.

Several limitations of this study must be acknowledged. The cohort was drawn from a single centre, and with 135 participants, the stability and external generalisability of the ROC-derived thresholds is uncertain. No multivariate analysis was performed; the confounding effects of age, abstinence duration, sperm concentration, and motility were not controlled, and the independent predictive value of each index for DFI therefore remains unknown. DNA fragmentation was assessed using a single assay platform, and the derived thresholds may not translate directly to TUNEL- or SCSA-based DFI measurements, which capture different dimensions of DNA damage. Morphological scoring, despite adherence to WHO criteria and inter-observer cross-validation, retains a subjective element, and variability between laboratories and between conventional and automated morphometry systems is well documented. Additionally, although the minimum cell counts used for morphology assessment (200 spermatozoa) and DNA fragmentation analysis (500 spermatozoa) comply with WHO (2021) recommendations and standard sperm chromatin dispersion protocols, these sample sizes may introduce sampling variability at the level of individual cell classification. Because TZI, SDI, and MAI are composite indices derived from per-cell morphological classification, small differences in the categorisation of a limited number of spermatozoa could influence the resulting index values and, in turn, the stability of the derived ROC thresholds. Similarly, DFI estimates based on 500 cells, while methodologically acceptable, may contribute additional uncertainty to threshold derivation. Future validation studies should consider whether larger per-sample cell counts improve the reproducibility and external validity of the proposed thresholds.

Mean sexual abstinence duration in this cohort was 4.53 days, within the WHO-recommended range, and is therefore unlikely to have introduced significant variability in semen parameters across the study population. However, occupational exposure to gonadotoxic agents or environmental toxicants was not formally assessed and remains an unmeasured potential confounder. The study did not include oxidative stress markers, chromatin maturity assays, or clinical reproductive outcomes, limiting contextualisation of these findings within a broader male fertility assessment framework. The absence of comparator biomarkers — including oxidative stress markers such as reactive oxygen species, chromatin maturity indices, and protamine deficiency assays — means that the independent predictive contribution of morphological indices relative to other established predictors of sperm DNA damage cannot be determined from the present data. This represents a priority for future multivariate studies incorporating a broader panel of sperm function biomarkers.

Future work should address these limitations through prospective, multi-centre studies with larger sample sizes, multivariate modelling incorporating established confounders, and direct comparison of morphological index performance against other predictors of chromatin integrity. Integration of clinical outcome data — natural conception rates and ART cycle results — would provide a more meaningful benchmark than the surrogate DFI endpoint employed here. Until such external validation is available, the thresholds reported in this study should be treated as exploratory reference values rather than established clinical cut-offs, and their application limited to an adjunctive role within a comprehensive semen analysis framework.

## Conclusion

TZI, SDI, and MAI demonstrated weak but statistically significant correlations with sperm DNA fragmentation in this single-centre cohort. At Youden-optimised thresholds of TZI > 1.85, SDI > 2.42, and MAI > 2.53, all three indices achieved high sensitivity (82–90%) but consistently low specificity (44–53%) for identifying men with DFI > 20%, reflecting a screening-type rather than diagnostic performance profile. Among the three, MAI showed the strongest association with DFI and the highest sensitivity, though the differences between indices were modest. These findings suggest that morphological indices may provide preliminary information to identify men who could benefit from direct DNA fragmentation assessment, particularly in settings where access to functional testing is inconsistent. These thresholds should not be used to guide ART treatment decisions or replace direct DFI testing under any circumstances. However, given the exploratory nature of this study, the modest discriminatory performance observed, and the inherent subjectivity of morphological assessment, these thresholds require prospective validation in larger, independent, multi-centre cohorts before any clinical implementation can be considered. Direct DNA fragmentation testing remains the reference standard and should not be deferred on the basis of morphological index values alone.

## Data Availability

The data that support the findings of this study are available from University of Debrecen but restrictions apply to the availability of these data, which were used under license for the current study, and so are not publicly available. Data are however available from the authors upon reasonable request and with permission of University of Debrecen.
